# Concomitant Langerhans cell histiocytosis of cervical lymph nodes in adult patients with papillary thyroid carcinoma: A report of two cases and review of the literature

**DOI:** 10.4322/acr.2021.253

**Published:** 2021-03-12

**Authors:** Bayan Maraqa, Maxim Al-Ashhab, Nazmi Kamal, Mousa El Khaldi, Maher Sughayer

**Affiliations:** 1 King Hussein Cancer Center, Department of Pathology and Laboratory Medicine, Amman, Jordan; 2 King Hussein Cancer Center, Department of Radiology, Amman, Jordan

**Keywords:** Langerhans cell histiocytosis, papillary thyroid carcinoma, adults, BRAF

## Abstract

**Objective:**

: Langerhans cell histiocytosis (LCH) is an uncommon entity of unknown etiology. It contains a wide range of clinical presentations. The discovery of oncogenic *BRAF V600E* mutation in LCH has provided additional evidence that LCH is a neoplasm. Papillary thyroid carcinoma is the most common cancer of the thyroid characterized by a high incidence of *BRAF V600E* mutations. LCH with concomitant PTC is rare, with few cases reported in the literature.

**Cases summary:**

We identified two cases of LCH with concomitant papillary thyroid carcinoma in adult patients. The first was a 49-year-old female with a thyroid nodule diagnosed with papillary thyroid carcinoma. Later, the patient had a left neck mass; Ultrasound-guided lymph node FNA was diagnosed with Langerhans histiocytosis. Subsequently, a chest CT scan revealed signs of Langerhans cell histiocytosis in the lung. The second case refers to a 69-year-old male who presented with a left thyroid nodule diagnosed on FNA cytology as papillary thyroid carcinoma. The patient was found to have multiple bone lytic lesions. Biopsies revealed Langerhans cell histiocytosis. Later, the patient experienced LCH involvement of the bone marrow with associated secondary myelofibrosis.

**Conclusions:**

LCH is rare in adults; the association with papillary thyroid carcinoma is reported and should be considered in the presence of Langerhans cell groups along with PTC, whether in the thyroid gland or cervical lymph nodes. Once LCH has been diagnosed, pulmonary involvement should also be investigated. This will direct treatment plans for patients with pulmonary or systemic disease involvement.

## INTRODUCTION

Langerhans cell histiocytosis (LCH) involves various clinical disorders that share Langerhans cells' proliferation with typical morphology, immunophenotype, and ultrastructural characteristics.^1^ The estimated annual incidence is approximately 5 cases per 1 million population, with most cases occurring in childhood.^2^ For therapeutic purposes, patterns of involvement are typically stratified into single-system LCH and multisystem LCH.^3^

As Langerhans cell histiocytosis (LCH) is rare in adults (detected in one to two adults per 1 million populations),^4^ it is challenging to associate clinical features with prognosis, ideal treatment, and a usual history.^5^ In comparison to childhood LCH, there is a lack of explicit evidence-based references and recommendations. Unlike childhood LCH, a rapidly progressive form is typically not observed in adults. The usual presentation is with a unifocal disease, most often a lytic bone lesion or solitary lesions at other sites with enlarged lymph nodes.^6^

Isolated lung involvement is a special type of LCH that almost always occurs in adult smokers in their third and fourth decades. While adult LCH is sometimes considered a pulmonary disorder, it may present with isolated extrapulmonary manifestations.^5^ The most common non-pulmonary locations include the bone, skin, and pituitary gland and less frequently the lymph nodes, liver, spleen, gut, and central nervous system (CNS).^6^ The bone marrow is rarely involved in adults, as opposed to children.^7^ The thyroid gland may occasionally be involved in LCH, but isolated thyroid LCH is extremely rare. Thyroid involvement is more frequently reported in adults than in children with a slight female predominance.^8^

While papillary thyroid carcinoma (PTC) is the most common cancer of the thyroid gland, these two entities' co-existence in the thyroid gland is unusual and reported only in a few cases available in the literature.

Herein, we report two cases of LCH with concomitant papillary thyroid carcinoma in adult patients.

## MATERIAL AND METHODS

In the Department of Pathology at the King Hussein Cancer Center, we identified two Langerhans cell histiocytosis cases in conjunction with papillary thyroid carcinoma. Preoperative thyroid ultrasound and cytology FNA smears were available in each case. The excised tissue was serially sectioned and fixed in 10% buffered formalin overnight. The sections taken were routinely processed for paraffin embedding and were stained using hematoxylin and eosin. Immunostaining was performed on the paraffin-embedded material using the avidin-biotin complex protocol with an iVIEW DAB detection kit (Ventana Medical Systems). Monoclonal antibodies against CDIa (EP3622,Ventana), S100 (Polyclonal, Ventana) and CD68 (KP-1,Ventana) were used in both cases. All immunostains were performed on the Ventana Benchmark XT automated immunostainer.

We conducted a literature review via PubMed. We searched for the English language published case series and case reports on adults with concomitant Langerhans cell histiocytosis and papillary thyroid carcinoma. The year of publication was limited to 1990 onwards.

## CASE 1

A 49-year-old female patient was diagnosed with a thyroid nodule on routine neck ultrasound; she had no family history of thyroid cancer. Laboratory tests showed euthyroidism. Neck ultrasound revealed partly cystic and hypoechoic nodules in both thyroid lobes, the largest of which was 10 x 8 x 15 mm in the right lobe. No enlarged neck lymph nodes were identified.

**Figure 1:**
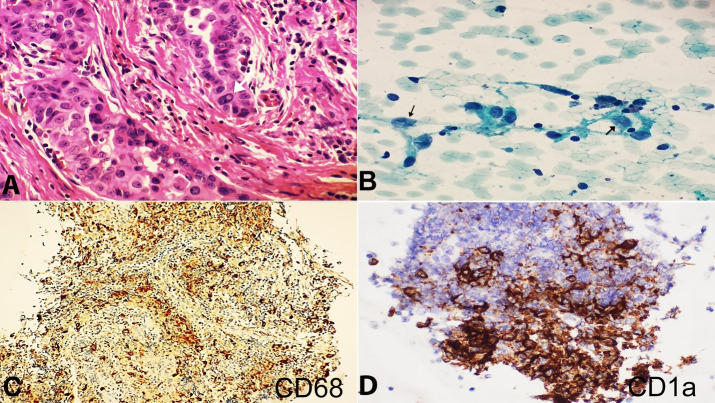
**A –** Thyroid gland with classic papillary thyroid carcinoma (H&E,40x); **B –** Cytologic preparation showing cells with reniform nuclei and abundant eosinophilic cytoplasm, the nuclei show prominent grooving (Arrows) (PAP stain 40x); **C and D –** Positivity for CD68 and CD1a immunostains, respectively done on the cell block (20x).

Ultrasound-guided FNA was performed and interpreted as suspicious for papillary thyroid carcinoma, and a total thyroidectomy was undertaken. The thyroid gland was received intact. Serial sectioning revealed multiple nodules. The tumor was identified in the right lobe, measuring 0.3x0.2x0.1cm. Microscopic examination revealed classic papillary thyroid microcarcinoma in multi-nodular goiter (Figure 1A).

The patient was discharged after a week and scheduled for close follow-up.

Five months later, the patient presented with odynophagia. Physical examination showed a left neck mass, which on the ultrasound examination consisted of an enlarged rounded cortically thickened left level II lymph node measuring 1.5 x 2.5 cm, likely metastatic, with few smaller yet pathological left level Ib and II lymph nodes. Ultrasound-guided lymph node FNA was done. The smears showed cells with reniform nuclei and abundant eosinophilic cytoplasm. The nuclei displayed prominent grooving but no nuclear pseudoinclusions (Figure 1B). Many eosinophils were seen in the background. These cells were positive for CD68 and CD1a (Figure 11D) and negative for S100, ERG, and TTF1.

**Figure 2:**
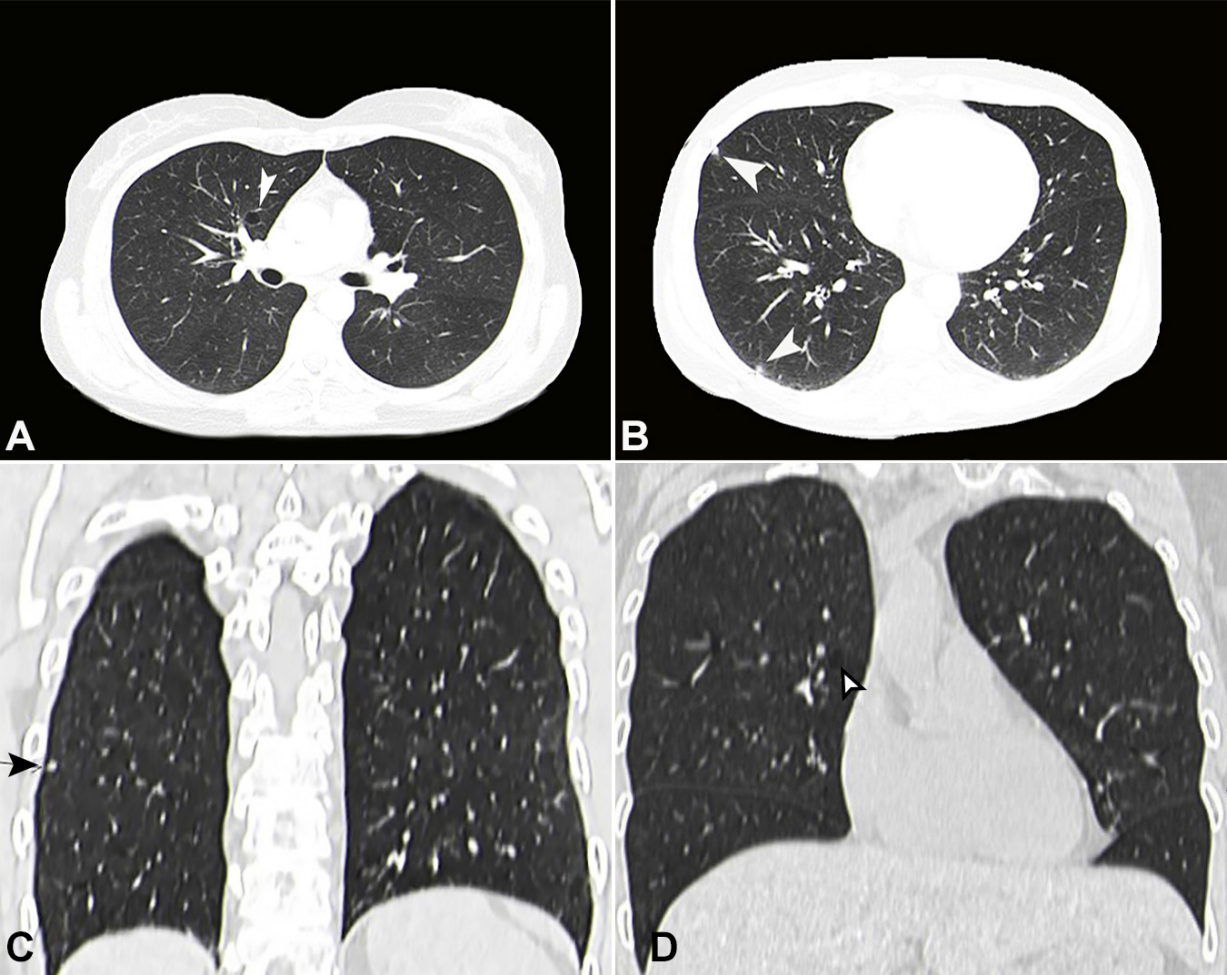
Chest CT scan, **A and B –** axial plane- with tiny pulmonary nodules and cysts (arrows) involving both lungs, likely due to pulmonary Langerhans cell histiocytosis. **C and D -** Coronal plane - showing pulmonary cysts and nodules.

The diagnosis was consistent with Langerhans cell histiocytosis. Subsequently, a chest CT scan was performed and revealed small pulmonary nodules and cysts affecting both lungs, primarily seen in the lower lobes, likely due to pulmonary Langerhans cell histiocytosis (Figure 2). After three months, the Chest CT scan showed unchanged bilateral pulmonary nodules with no mediastinal or axillary lymphadenopathy, and the bone scan revealed nonspecific lytic lesions within the proximal right femoral shaft. The patient was asked to stop smoking, and she was scheduled for a regular follow up CT scan every three months.

## CASE 2

A 69-year-old male presented a left thyroid nodule diagnosed on FNA cytology as papillary thyroid carcinoma. During investigations, the patient was found to have multiple bone lytic lesions. Biopsies of the lumbar vertebra and left clavicle showed Langerhans cell histiocytosis. The patient underwent total thyroidectomy with left cervical lymph node dissection. Sectioning of the specimen revealed two lesions in the right thyroid lobe measuring 3.7 X 3.5 X 2.5 cm and 0.7 X 0.7 X 0.5 cm and a single lesion in the left thyroid lobe measuring 1 X 0.7 X 0.7 cm.

Additionally, the left thyroid lobe showed a goitrous nodule measuring 3.7 X 3.5 X 2.5cm. The microscopic examination concluded a multifocal classical papillary carcinoma confined to the thyroid gland with no extra-capsular extension neither lymph node involvement. However, four lymph nodes showed morphologic features of focal involvement by Langerhans cell histiocytosis. Immunohistochemical stains have confirmed these features. The Langerhans cells were positive for CD1a and negative for TTF-1. The thyroid gland was extensively examined without any morphological evidence of Langerhans cell histiocytosis involvement.

**Figure 3:**
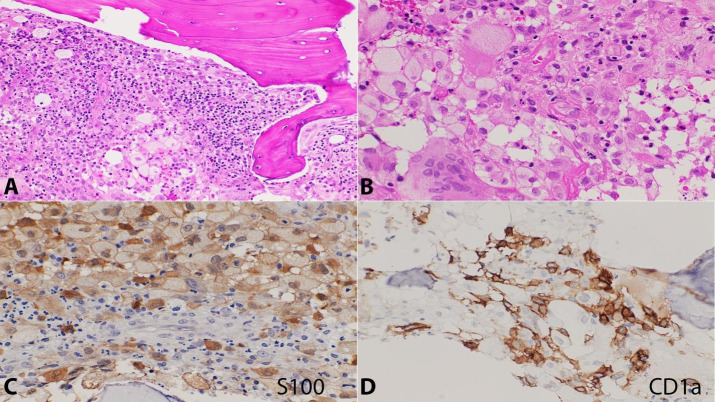
**A and B –** Bone marrow biopsy with multifocal lesions composed of histiocytic cells with grooved nuclei admixed with multinucleated giant cells and foamy histiocytes in the background with eosinophils and plasma cells (20x and 40x). The histiocytic cells are immunoreactive for S100 and CD1a in **C and D**, respectively (40x).

Four years later, the patient presented with anemia leukocytosis, and bone marrow biopsy showed marrow involvement by Langerhans cell histiocytosis (Figure 3). The estimated degree of involvement was 40% with associated secondary myelofibrosis.

## DISCUSSION

Langerhans cell histiocytosis (LCH) is an uncommon entity with unknown etiology. The question of whether LCH is a neoplastic or a reactive process has long been debated. Evidence of clonality in LCH was reported more than 20 years ago, supporting the idea that LCH is a neoplastic process.^9^ Recently, the discovery of oncogenic *BRAF V600E* mutation in 25 to 64 percent of LCH cases has provided additional evidence that LCH is neoplasm.^10^^-^^13^ While the presence of *BRAF V600E* in LCH is associated with an increased risk of recurrence, there does not appear to be a correlation between *BRAF* mutation and survival time.^11^

LCH identification should be based on the histological and immunophenotypic characteristics of the lesion. The basic standard for the diagnosis is the cytological identification of the typical LCH cells that exhibit CD1a positivity.^6^^,^^14^ Additional workups may include detection of mutations of the *BRAF-ERK* pathway, which may provide more therapeutic options for refractory and recurrent diseases.^6^^,^^15^

It is such a rare entity in adults, making a robust evidence-based guideline and references lack.^4^ Also, the clinical development of LCH in adults may vary from self-limiting to chronic recurrent disease, which makes it necessary to expand studies and research on this group of patients.

The number of adult patients affected is likely to be underestimated, as specialist treatment facilities are rarely contacted in cases of advanced or recurrent disease.^16^ Besides, several cases are considered incidental, found during the investigation for other reasons, as shown in our two adult cases.

Papillary thyroid carcinoma (PTC) is the most common thyroid cancer, accounting for 80%-90% of thyroid carcinoma cases. It generally shows an excellent prognosis with a 5-year survival rate of almost 100%.^17^

Thyroid or cervical lymph node involvement by LCH in the presence of co-existing papillary thyroid carcinoma is rare; only 16 cases have been reported in English literature (Table 1).^18^^-^^30^

**Table 1:** Summary of the literature review of reported cases of Concomitant Langerhans cell histiocytosis and papillary thyroid carcinoma in adult patients

Author	Gender	Age	PTC and LCH in thyroid	LCH in lymph nodes	Involvement of other organs
Goldstein N et al.^18^	Female	31	Yes	Not described	Bone, Pituitary gland, Lung, Skin, Vagina
Safali et al.^19^	Male	51	Yes	Yes	No
Saiz et al.^20^	Male	43	Yes	Not described	No
Foulet-Roge et al.^21^	Female	42	Yes	Not described	No
Jamaati et al.	Male	24	Yes	Not described	Lung
Vergez et al.^23^	Male	29	Yes	Not described	Bone, Pituitary gland, Lung, Skin
Chung DH et al.^24^	Female	53	Yes	Not described	Not reported
Ceyran et al.^25^	Male	37	Yes	Yes	Not reported
Gordon et al.^26^	Female	23	Yes	Not described	Labia Vulva
Alzahrani et al.^27^	Female	27	Yes	Yes	No
Wu et al.^28^	Male	40	Yes	Yes	Lung, Liver
Hamad et al.^29^	Female	37	Yes	Yes	No
Ozisik et al.^30^	Male	58	Yes	Not described	Pituitary gland
	Male	45	Yes	Yes	Pituitary gland, gingiva
Current Case 1	Female	49	PTC only	Yes	Lung
Current Case 2	Male	69	PTC only	Yes	Bone, bone marrow

Two cases have been excluded; one is a child, and in the second case, papillary thyroid carcinoma was not reported.^31^^,^^32^ Seven cases have evidence of LCH in other organs, most often in the lung and pituitary gland. Bone involvement has been confirmed in two cases. The presence of lymph node involvement was seen in six cases. Our two cases have papillary thyroid carcinoma and evidence of LCH in the cervical lymph nodes and a second location each (lung or bone). LCH occurring in lymph nodes that drain solid malignant tumors such as melanoma and papillary thyroid carcinoma have been reported. However, it was typically associated with concomitant metastasis in the same lymph node.^33^^,^^34^^,^^36^ In fact, this will make the diagnosis more challenging, as cells seen in LCH can mimic those seen in PTC and malignant melanoma; moreover, both malignant melanoma and LCH show S100 protein-positive reaction. In both of our cases, there was no evidence of metastases in lymph nodes. One case had features of probable pulmonary involvement in the CT scan, and the other case also displayed involvement of bone and bone marrow, suggesting systemic disease.

Our two adult cases’ importance relies on the confirmation of LCH involvement of cervical lymph nodes without evidence of primary thyroid disease. Our vision is that LCH can involve the cervical lymph nodes as part of disseminated disease.

Additionally, several studies have shown a higher prevalence of hematological and solid malignancies among LCH patients.^35^^,^^36^ A study by Jennifer Ma et al.^36^ of adult LCH patients found an exceptionally high number of additional malignancies. The identified malignancies were diagnosed either earlier or at the same time as a diagnosis of LCH, indicating that the cause of malignancies is not secondary to LCH treatment. Similarly, in our two cases, the patients did not receive any additional treatment, and the two entities appeared within a short period.

Papillary thyroid carcinoma is characterized by a high rate of *BRAF V600E* mutations.^37^ Yet, the prognostic significance of these mutations remains controversial.^38^ *BRAF* is a protein kinase involved in cellular processes, including cell survival, proliferation, and differentiation. The *BRAF* gene mutations are seen in many cancers, among which the *BRAF V600E* is the most common mutation.^39^ The association of LCH with PTC can be attributed to the fact that the *BRAF* mutation may play a role in both diseases' underlying pathogenesis. However, there is no sufficient available data regarding the *BRAF* mutation status in cases of co-existing LCH and PTC in the literature, and it is not available in both of our cases. Hamad et al.^29^ found *BRAF V600E* and *BRAF V600K* mutations in PTC and LCH tissues, respectively. While Ozisik et al.^30^ reported *BRAF V600E* in both PTC and LCH tissues. These two lesions co-exist in the thyroid gland, indicating an etiological relationship between the two disorders.

Isolated involvement of lymph nodes by LCH is rare, but spontaneous regressions have been observed. As a result, watch and wait in adults with LCH may be acceptable for isolated lymph node involvement.^6^^,^^40^ Therefore, once LCH is diagnosed in a lymph node, the presence of other lymph node groups or pulmonary involvement should be investigated. This will guide therapeutic management strategies to such patients with pulmonary involvement or systemic disease.

One important finding in our cases is the ability to diagnose LCH on FNA. In the first case, cytological examination, when combined with immunohistochemistry on the cell-block (CB), is sufficient for confirming the diagnosis of Langerhans cell histiocytosis.

Our findings suggest that the emergence of other tumors such as papillary thyroid carcinoma should be expected in patients with Langerhans cell histiocytosis. Similarly, lymphadenopathy in patients with papillary thyroid carcinoma may not necessarily be a metastatic tumor, particularly when combined with lesions elsewhere in the body. In this setting, the pathological examination of the biopsy specimens or FNA cytology distinguishes between the two conditions.

## CONCLUSIONS

LCH in adults is rare. Association with papillary thyroid carcinoma is rarely seen and should be considered in the presence of Langerhans cell groups along with PTC, whether in the thyroid gland or cervical lymph nodes. Once LCH has been diagnosed, pulmonary involvement should also be examined. This will direct treatment plans for patients with pulmonary or systemic disease involvement.

We believe that the pathologist and clinician need a greater understanding of LCH and its various clinical manifestations in adult patients to ensure that the diagnosis can be made.

## References

[1] Harmon CM, Brown N (2015). Langerhans cell histiocytosis: A clinicopathologic review and molecular pathogenetic update. Arch Pathol Lab Med.

[2] Swerdlow S, Campo E, Harris NL (2017). WHO classification of tumours of haematopoietic and lymphoid tissues.

[3] Orkin SH, Nathan DG, Ginsburg D, Look AT, Fisher DE, Lux S (2014). Nathan and Oski's hematology and oncology of infancy and childhood.

[4] Choi JE, Lee HR, Ohn JH (2014). Adult multisystem langerhans cell histiocytosis presenting with central diabetes insipidus successfully treated with chemotherapy. Endocrinol Metab (Seoul).

[5] Peters GE, Harder SL, Fladeland D, Ward HA (2009). Occult presentation of adult Langerhans Cell Histiocytosis with extrapulmonic manifestations. Chest.

[6] Girschikofsky M, Tazi A (2018). Adult langerhans cell histiocytosis. Histiocytic Disorders.

[7] Minkov M, Pötschger U, Grois N, Gadner H, Dworzak MN (2007). Bone marrow assessment in Langerhans cell histiocytosis. Pediatr Blood Cancer.

[8] Attakkil A, Thorawade V, Jagade M, Kar R, Parelkar K (2015). Isolated Langerhans histiocytosis in thyroid: thyroidectomy or chemotherapy?. J Clin Diagn Res.

[9] Willman CL, Busque L, Griffith BB (1994). Langerhans’-cell histiocytosis (histiocytosis X)--A clonal proliferative disease. N Engl J Med.

[10] Badalian-Very G, Vergilio JA, Degar BA (2010). Recurrent BRAF mutations in Langerhans cell histiocytosis. Blood.

[11] Berres ML, Lim KP, Peters T (2014). BRAF-V600E expression in precursor versus differentiated dendritic cells defines clinically distinct LCH risk groups. J Exp Med.

[12] Go H, Jeon YK, Huh J (2014). Frequent detection of BRAFV 600E mutations in histiocytic and dendritic cell neoplasms. Histopathology.

[13] Roden AC, Hu X, Kip S (2014). BRAF V600E expression in Langerhans cell histiocytosis: clinical and immunohistochemical study on 25 pulmonary and 54 extrapulmonary cases. Am J Surg Pathol.

[14] Girschikofsky M, Arico M, Castillo D (2013). Management of adult patients with Langerhans cell histiocytosis: recommendations from an expert panel on behalf of Euro-Histio-Net. Orphanet J Rare Dis.

[15] Hyman DM, Puzanov I, Subbiah V (2015). Vemurafenib in multiple nonmelanoma cancers with BRAF V600 Mutations. N Engl J Med.

[16] Aricò M, Girschikofsky M, Généreau T, Klersy C, McClain K, Grois N (2003). Langerhans cell histiocytosis in adults. Report from the International Registry of the Histiocyte Society. Eur J Cancer.

[17] Czarniecka A, Oczko-Wojciechowska M, Barczyński M (2016). BRAF V600E mutation in prognostication of papillary thyroid cancer (PTC) recurrence. Gland Surg.

[18] Goldstein N, Layfield LJ (1991). Thyromegaly secondary to simultaneous papillary carcinoma and histiocytosis X. Report of a case and review of the literature. Acta Cytol.

[19] Safali M, McCutcheon JM, Wright DH (1997). Langerhans cell histiocytosis of lymph nodes: draining a papillary carcinoma of the thyroid. Histopathology.

[20] Saiz E, Bakotic BW (2000). Isolated Langerhans cell histiocytosis of the thyroid: a report of two cases with nuclear imaging-pathologic correlation. Ann Diagn Pathol.

[21] Foulet-Rogé A, Josselin N, Guyetant S (2002). Incidental langerhans cell histiocytosis of thyroid: case report and review of the literature. Endocr Pathol.

[22] Jamaati HR, Shadmehr MB, Saidi B, Khosravi A, Arab M, Mohammadi F (2009). Langerhans cell histiocytosis of the lung and thyroid, co-existing with papillary thyroid cancer. Endocr Pathol.

[23] Vergez S, Rouquette I, Ancey M, Serrano E, Caron P (2010). Langerhans cell histiocytosis of the thyroid is a rare entity, but an association with a papillary thyroid carcinoma is often described. Endocr Pathol.

[24] Chung DH, Ha SY, Cho HY (2012). Langerhans cell histiocytosis in the thyroid and draining lymph nodes: a case report. Endocrinol Metab (Seoul).

[25] Ceyran AB, Senol S, Bayraktar B, Ozkanlı S, Cinel ZL, Aydın A (2014). Langerhans cell histiocytosis of the thyroid with multiple cervical lymph node involvement accompanying metastatic thyroid papillary carcinoma. Case Rep Pathol.

[26] Gordon MS, Gordon MB (2016). Occult Langerhans cell Histiocytosis presenting with papillary thyroid carcinoma, a thickened pituitary stalk and diabetes insipidus. Case Rep Endocrinol..

[27] AlZahrani R, Algarni M, Alhakami H (2016). Thyroid Langerhans cell histiocytosis and papillary thyroid carcinoma. Gland Surg.

[28] Wu X, Chen S, Zhang LY, Luo YP, Jiang Y, Feng RE (2017). Langerhans cell histiocytosis of the thyroid complicated by papillary thyroid carcinoma: A case report and brief literature review. Medicine (Baltimore).

[29] Al Hamad MA, Albisher HM, Al Saeed WR, Almumtin AT, Allabbad FM, Shawarby MA (2019). BRAF gene mutations in synchronous papillary thyroid carcinoma and Langerhans cell histiocytosis co-existing in the thyroid gland: a case report and literature review. BMC Cancer.

[30] Ozisik H, Yurekli BS, Demir D (2020). Langerhans cell histiocytosis of the thyroid together with papillary thyroid carcinoma. Hormones (Athens).

[31] Burnett A, Carney D, Mukhopadhyay S, Scalzetti EM, Leino D, Souid AK (2008). Thyroid involvement with Langerhans cell histiocytosis in a 3-year-old male. Pediatr Blood Cancer.

[32] Thompson LD (1996). Langerhans cell histiocytosis isolated to the thyroid gland. European archives of oto-rhino-laryngology: official journal of the European Federation of Oto-Rhino-Laryngological Societies (EUFOS): Affiliated with the German Society for Oto-Rhino-Laryngology. Head Neck Surg.

[33] Schofield JB, Alsanjari NA, Davis J, MacLennan KA (1992). Eosinophilic granuloma of lymph nodes associated with metastatic papillary carcinoma of the thyroid. Histopathology.

[34] Richmond I, Eyden BP, Banerjee SS (1995). Intranodal Langerhans’ cell histiocytosis associated with malignant melanoma. Histopathology.

[35] Egeler RM, Neglia JP, Puccetti DM, Brennan CA, Nesbit ME (1993). Association of Langerhans cell histiocytosis with malignant neoplasms. Cancer.

[36] Ma J, Laird JH, Chau KW, Chelius MR, Lok BH (2019). Langerhans cell histiocytosis in adults is associated with a high prevalence of hematologic and solid malignancies. Cancer Med..

[37] Xing M (2007). BRAF mutation in papillary thyroid cancer: pathogenic role, molecular bases, and clinical implications. Endocr Rev.

[38] Lloyd RVOR, Klöppel G, Rosai J (2017). WHO classification of tumours of endocrine organs.

[39] Davies H, Bignell GR, Cox C (2002). Mutations of the BRAF gene in human cancer. Nature.

[40] Lo WC, Chen CC, Tsai CC, Cheng PW (2009). Isolated adult Langerhans’ cell histiocytosis in cervical lymph nodes: should it be treated?. J Laryngol Otol.

